# Integrated Nd:YAG–Er:YAG–SWEEPS Laser Approach for Maxillary Dental Trauma in an Anticoagulated Geriatric Patient

**DOI:** 10.1155/crid/8134806

**Published:** 2026-10-05

**Authors:** Ilaria Giovannacci, Beatrice Spaggiari, Rita Antonelli, Marco Meleti, Paolo Vescovi

**Affiliations:** ^1^ Department of Medicine and of Surgery University of Parma Parma Italy unipr.it

**Keywords:** case report, dental trauma, Er:YAG laser, geriatric patient, Nd:YAG laser, SWEEPS

## Abstract

Traumatic dental injuries (TDIs) in older adults present complex clinical and public health challenges because of frailty, multimorbidity, and antithrombotic therapy. Invasive surgery in anticoagulated patients increases the risk of hemorrhage and delayed healing. Multiwavelength laser systems provide a minimally invasive alternative, combining precise ablation, hemostasis, and disinfection. A 92‐year‐old woman receiving anticoagulant therapy for atrial fibrillation presented with multiple anterior maxillary fractures following a fall. The right maxillary central incisor (Tooth 11) showed a complete coronal fracture with pulp exposure (Ellis Class 3) and a palatal fracture line extending below the bone crest. Owing to systemic risk, a tooth‐preserving laser‐assisted approach was adopted instead of extraction. Gingivectomy was performed using an Nd:YAG laser (1064 nm; 3.5 W, 60 Hz, fluence: 72.5 J/cm^2^, power density: 4.36 kW/cm^2^) to achieve hemostasis and soft‐tissue decontamination. Bone vaporization was completed with an Er:YAG laser (2940 nm; QSP mode, H14 handpiece; 250 mJ, 15 Hz) to expose the fracture margin. Root‐canal decontamination used the Er:YAG SWEEPS technique for photoacoustic activation of irrigants (NaOCl: 5%, EDTA: 17%), enhancing biofilm removal and smear‐layer disruption. The tooth was reconstructed with fiber‐post and composite resin buildup, followed by monolithic zirconia crowns on both central incisors. Soft‐tissue healing was complete at 15 days, and functional rehabilitation was achieved without complications. Two‐year clinical and radiographic follow‐up confirmed stable periapical conditions, preserved gingival contours, and satisfactory aesthetics. The coordinated use of Nd:YAG, Er:YAG, and Er:YAG SWEEPS lasers enabled selective tissue management—hemostasis, bone remodeling, and endodontic disinfection—under continuous anticoagulation, avoiding extraction and minimizing operative trauma. Rapid soft‐tissue healing was observed clinically during follow‐up. Within the limitations of a single case report, multiwavelength laser‐assisted therapy may represent a conservative treatment option for managing dental trauma in anticoagulated geriatric patients, supporting tooth preservation and functional rehabilitation.

## 1. Background

Traumatic dental injuries (TDIs) in older adults represent a considerable clinical and public health challenge due to the combination of age‐related frailty, comorbidities, and complex pharmacological profiles. The impact of trauma among geriatric patients has increased notably over the past two decades as life expectancy rises and falls continue to be the leading cause of accidental injury in older adults. According to guidelines 2023 World Society of Emergency Surgery (WSES) for elderly trauma, falls account for over 70% of maxillofacial injuries in individuals above the age of 65, with markedly higher morbidity and delayed recovery compared with younger cohorts [1, 2].

Aging introduces structural and physiological changes—reduced vascular elasticity, limited tissue perfusion, and altered immune responses—that collectively impair wound healing, prolong inflammation, and diminish regenerative potential [3]. Moreover, frailty, characterized by reduced homeostatic reserve, affects over 40% of individuals, often overlapping with multimorbidity [4, 5]. These conditions significantly complicate perioperative management following oral or dental trauma.

A further challenge derives from polypharmacy and antithrombotic therapy. It is estimated that more than 60% of patients aged > 65 years regularly take five or more systemic drugs, whereas nearly 20% take 10 or more (hyperpolypharmacy) to control cardiovascular, metabolic, or rheumatologic disorders [6]. Among these, anticoagulant or antiplatelet agents are particularly critical, being prescribed in 35%–40% of older adults [7]. The hemostatic imbalance caused by these therapies complicates routine dental interventions, rendering traditional surgical or flap‐based approaches risky because of uncontrolled bleeding, hematoma formation, and delayed wound closure.

In older adults, TDIs are not only frequent but also clinically significant because they may compromise function, nutrition, communication, and quality of life, whereas their management is often complicated by multimorbidity, frailty, and anticoagulant therapy.

In this context, minimally invasive tooth‐preserving strategies are particularly relevant, as they may reduce surgical burden while facilitating bleeding control and postoperative healing in medically compromised older patients.

Clinicians thus face a scenario where invasive methods must be minimized without compromising decontamination or tissue management. In medically compromised older adults, multimodal laser therapy may offer a minimally invasive option for soft‐tissue management, hard‐tissue remodeling, and adjunctive endodontic disinfection. The Nd:YAG (neodymium‐doped: yttrium‐aluminum‐garnet) laser (1064 nm) offers deep yet selective absorption in hemoglobin, enabling rapid coagulation of small vessels and effective bacterial reduction. The Er:YAG (erbium‐doped: yttrium‐aluminum‐garnet) laser (2940 nm), with strong affinity for water and hydroxyapatite, facilitates conservative removal of contaminated or necrotic bone with minimal thermal insult, critical in elderly bone with decreased vascular supply. The Er:YAG‐SWEEPS (shock wave‐enhanced emission photoacoustic streaming) technique extends these benefits to endodontic therapy by generating high‐frequency shock waves that increase irrigant activation, biofilm disruption, and ion penetration within the root canal [8].

This integrated laser approach—combining Nd:YAG for hemostasis and soft‐tissue decontamination, Er:YAG for atraumatic bone remodeling, and Er:YAG‐SWEEPS for acoustic endodontic cleaning—offers a coherent, minimally invasive solution to the multifactorial challenges of managing TDIs in frail, polymedicated, and anticoagulated geriatric patients.

This case report is aimed at describing the conservative management of a complicated crown fracture with subgingival/subcrestal extension in an anticoagulated older adult using a multimodal laser‐assisted approach (Nd:YAG, Er:YAG, and Er:YAG‐SWEEPS), and to discuss its clinical rationale, operative sequence, and 2‐year outcome.

## 2. Case Presentation

A 92‐year‐old female patient with multiple comorbidities was referred to the emergency department after an accidental facial trauma from a fall. The patient′s medical history was significant for atrial fibrillation treated with warfarin (Coumadin), with regular cardiology follow‐up for anticoagulation monitoring, as well as hypertension, hypercholesterolemia and diabetes managed pharmacologically.

In view of the patient′s advanced age, multiple comorbidities, and ongoing anticoagulant therapy, treatment planning favored a minimally invasive tooth‐preserving approach whenever clinically feasible.

The patient was referred for management of multiple dental fractures in the maxillary anterior region, specifically teeth number 11, 12, 21, and 22. Fractures were graded according to the Ellis′ classification; in particular, Teeth 12, 21, and 22 sustained enamel and dentin fractures without pulp exposure (Class 2 trauma), whereas the right central incisor (Tooth 11) sustained a complete coronal fracture with pulp exposure (Class 3 trauma) [9]. Clinical examination showed no mobility of the residual root portion of Tooth 11 or of the other traumatized anterior teeth, a finding that supported the feasibility of a conservative treatment approach.

The most complex issue was certainly the one concerning the right central incisor. The tooth presented with a complete coronal fracture, pulp exposure, and a palatal fracture line extending beyond the bone crest (Figure 1A–E). The most plausible therapeutic indication was surgical extraction. However, given the patient′s comorbidities and ongoing anticoagulant therapy, it was decided to attempt tooth preservation, using a multimodal laser therapy for optimal tissue management, especially in complex patients. The patient agreed to the proposed treatment and provided written informed consent for treatment and publication. All data were collected in accordance with the Declaration of Helsinki.

**Figure 1:**
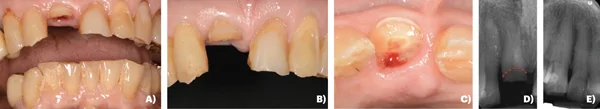
Photographs and x‐rays collection of the teeth affected by the traumatic injury. (A) General panoramic photograph of the oral cavity following trauma, buccal side. (B) Detail of the anterior maxillary group, showing the various coronal fractures of the four incisors. (C) Detail of the complete coronal fracture (Ellis Class 3) of the right upper central incisor. (D) Initial radiographic status of the right lateral and central upper incisors following impact trauma (Teeth 12 and 11). (E) Left lateral and central upper incisors following impact trauma (Teeth 21 and 22).

To perform root canal therapy (RCT) and reconstruction, it was necessary to expose the fracture line making the element isolable under rubber dam.

Gingivectomy was performed using neodymium laser (LightWalker, Ljubljana, Fotona, Slovenia) (parameter—output power: 3.5 W; frequency: 60 Hz; fiber diameter: 320 *μ*m; and power density: 4.36 kW/cm^2^; fluence: 72.5 J/cm^2^) (Figure 2A,B). By removing the palatal tissue adjacent to the fracture line, a further small, fractured root fragment was revealed and removed (Figure 2C).

**Figure 2:**
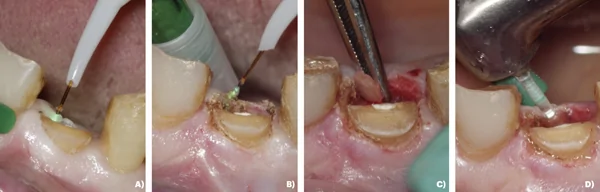
Minimally invasive surgical management of soft and hard tissues for exposure of the fracture margin. (A,B) Gingivectomy through Nd:YAG laser. (C) Additional tooth fragment found and removed. (D) Bone vaporization through Er:YAG laser.

The palatal fracture line was therefore even deeper, and it was necessary to perform bone vaporization to expose it. The Erbium laser (Er:YAG LightWalker, Ljubljana, Fotona, Slovenia) in QSP (quantum square pulse) mode was used to perform extremely targeted and minimally invasive bone vaporization (handpiece H14; QSP modality; parameter of −250 mJ; 15 Hz; fluence of 39 J/cm^2^; mean power density of ≈ 590 W/cm^2^) (Figure 2D). In this modality, each laser pulse is divided into five super‐short sub‐pulses separated by 85 *μ*s, a pulse structure intended to improve ablation efficiency while limiting interference from debris formation [10].

Once the fracture margin had been exposed, it was possible to achieve adequate isolation of the working field by placing a rubber dam (Figure 3A). At this stage, it was possible to proceed with the endodontic treatment, which was completed according to the standard procedure (shaping, irrigation and three‐dimensional filling of the root canal system). Once the working length (WL) had been determined using an apical locator (DentaPort ZX, Morita Europe, J Morita Corp.), the shaping phase followed, using nickel‐titanium (Ni‐Ti) rotary instruments (MTwo Sweden & Martina Spa, Italy, from 10.04 to 25.06).

**Figure 3:**
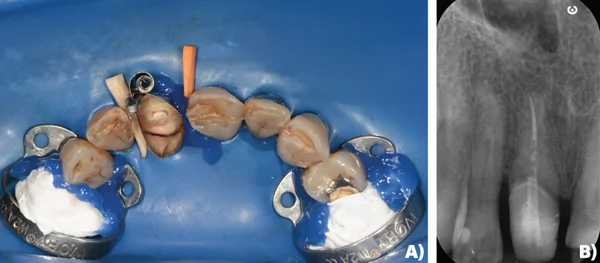
(A) Isolation of the working area with rubber dam. (B) Final x‐ray after RCT and fiber post reconstruction.

The decontamination of the endodontic system and smear layer removal were achieved through the use of irrigants, specifically 5% sodium hypochlorite (NaOCl) and 17% ethylenediaminetetraacetic acid (EDTA).

In order to enhance the effect of the irrigants, the SWEEPS technique was applied (Er:YAG laser, LightWalker, Fotona, Slovenia). This technology is based on the ability of the Er:YAG laser to generate shock waves that allow the irrigants to penetrate the dentinal tubules, inducing cavitation. It is used in the final stages of decontamination (immediately prior to root canal filling) to ensure a disinfection that is as close to sterility as possible. The specific sapphire tip (600 *μ*m, flat) was positioned inside the pulp chamber, and irradiation continued for 30 s, during which time a constant flow of irrigation fluid had to be maintained. The protocol consisted of three stages of laser application:

— 17% EDTA: 30 s of irradiation (15 Hz, 10 mJ, and 0.30 W) with continuous flow of EDTA, followed by a rinse with saline solution for about 1 min.
— 5% NaOCl: two continuous irradiations (15 Hz, 10 mJ, and 0.30 W) with NaOCl washes, each lasting 30 s, followed by a 1‐min rinse with saline solution.

Conventional irrigants such as NaOCl and EDTA are essential for root canal disinfection, although their effectiveness may be limited by contact time and the anatomical complexity of the canal system, particularly in areas that are difficult to instrument mechanically [11]. In this context, Er:YAG SWEEPS was used as an adjunctive strategy to enhance irrigant activation and improve decontamination within complex intracanal spaces, while maintaining a minimally invasive treatment approach [12].

The cleaned canal was ready for drying with sterile paper cones and for filling, achieved through continuous wave of condensation. During the same session, the tooth was reconstructed. Specifically, following a three‐step adhesive system (37% orthophosphoric acid, primer and adhesive OptiBond FL, Kerr Corp., Brea, California, United States) composite resin was applied for coronal core build‐up reconstruction (Enamel Plus, Micerium, Milan, Italy). As a last step a fiber post was cemented (Panavia V5 Universal system, Kuraray Noritake, Kuraray Europe GmbH, Germany) (Figure 3B). A fiber post was selected to support adhesive reconstruction of the severely compromised endodontically treated anterior tooth, owing to its favorable elastic behavior, stress distribution, and suitability for aesthetic coronal rehabilitation.

Preservation of Tooth 11 required careful periodontal and restorative assessment. In particular, maintenance of supracrestal tissue health, achievement of a clinically maintainable restorative margin, and the possibility of obtaining an adequate ferrule were considered essential prerequisites for long‐term rehabilitation. The concept of biological width, now more appropriately referred to as supracrestal tissue attachment, is of central importance when fracture margins extend subgingivally or subcrestally, as violation of this dimension may compromise periodontal stability and restorative success [13–15]. In the present case, soft‐ and hard‐tissue management was therefore directed toward exposing the fracture margin while preserving conditions compatible with periodontal health and subsequent prosthetic rehabilitation. The ferrule effect, defined as a circumferential band of sound tooth structure surrounding the post‐and‐core complex, is an important factor in the biomechanical performance of endodontically treated teeth [16]. In this case, a glass fiber post was selected because its elastic modulus is closer to that of dentin, allowing more favorable stress distribution and supporting adhesive reconstruction of a severely compromised anterior tooth. Together with ferrule‐oriented restorative planning, this choice was considered appropriate to improve biomechanical behavior and support the long‐term prognosis of the restored tooth. These considerations were integrated into the overall periodontal, functional, and prosthetic assessment in support of a conservative treatment plan.

At the 2‐week follow‐up, tissue healing was excellent (Figure 4A, B) but the left central incisor showed signs of pulpitis, necessitating root canal treatment. The clinical procedure followed for endodontic treatment was the same as that described above. The Er:YAG laser disinfection protocol was also executed in the same manner (as shown in Figure 5).

**Figure 4:**
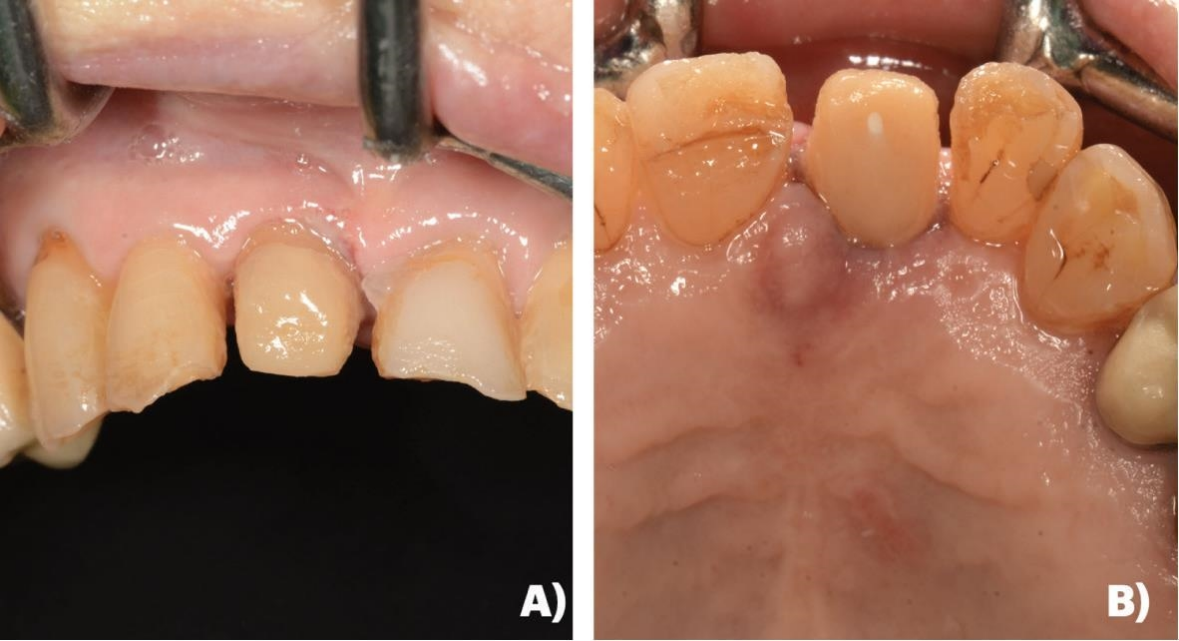
Soft‐tissue healing was complete at 15 days. Complete wound healing of the gingival margins. (A) Vestibular view and (B) palatal view.

**Figure 5:**
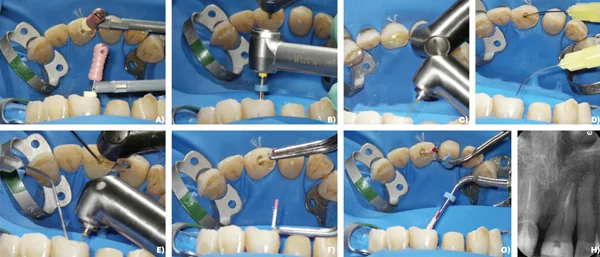
RCT of the left upper central incisor, summary panel of the different steps. (A) WL determination with apex locator. (B) Root canal shaping with Ni‐Ti rotary instruments. (C) Er:YAG irradiation (SWEEPS) with 17% EDTA. (D) 30‐s rinse with saline solution. (E) Er:YAG irradiation (SWEEPS) with 5% NaOCl—2 applications, 30 s each. (F) Canal drying with sterile paper cones. (G) Root canal filling with gutta‐percha. (H) Final x‐ray.

Once root canal treatment was completed, the old restorations in the coronal region were removed and the tooth was reconstructed by cementing a fiber post (Panavia V5 Universal system, Kuraray Noritake, Kuraray Europe GmbH, Germany). Simultaneously, the lateral incisors (Teeth 12–22) were restored directly with composite reconstructions (Figure 6A). Specifically, 37% orthophosphoric acid was applied for 30 s on enamel and 15 s on dentin (Figure 6B); after a thorough rinse a two‐step adhesive system (primer and adhesive OptiBond FL, Kerr Corp., Brea, California, United States) was employed. The adhesive was cured for 40 s on each tooth using an LED photopolymerization lamp in standard mode (1000 mW/cm^2^, Valo, Ultradent, Milan, Italy), and the composite material was layered (Enamel Plus, Micerium, Milan, Italy). (Figure 6C,D).

**Figure 6:**
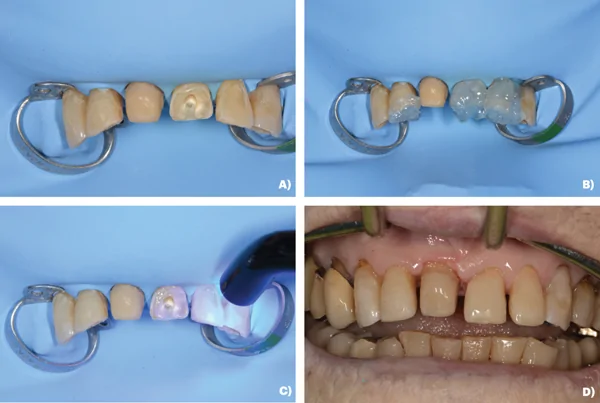
Coronal reconstruction of the left upper lateral incisor and the right upper incisors (central and lateral). (A) Teeth 12, 21, and 22 prior to restoration. (B) Application of 37% orthophosphoric acid. (C) Adhesive light‐curing. (D) Final photograph of the reconstructions.

At 4 weeks, the central incisors were considered ready for definitive prosthodontic rehabilitation. Treatment was initiated at that time because early soft‐tissue healing was favorable, the treated area appeared clinically stable, and timely coverage was considered important to protect the remaining tooth structure and restore function, particularly in view of the patient′s advanced age and overall frailty. A vertical finishing preparation was preferred (Figure 7A), followed by a final impression (Figure 7B) with digital optical scanning (Medit i500, Medit Corp, Seoul) and the fitting of resin temporary crowns with temporary cement (Temp‐Bond, Kerr Corp, California, United States). (Figure 7C).

**Figure 7:**
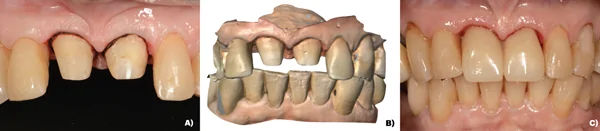
(A) Vertical finishing preparation of the upper central incisors. (B) Final digital impression. (C) Resin temporary crowns.

One week later, the final monolithic CAD‐CAM (computer‐aided design and computer‐aided manufacturing) zirconia crowns, manufactured in the laboratory (Figure 8), were fitted. Monolithic zirconia crowns were selected to provide mechanical reliability with conservative thickness requirements in the restoration of severely compromised anterior teeth. Although material selection in the anterior region requires careful aesthetic consideration, in the present case this option was considered appropriate in view of the limited remaining tooth structure, the patient’s functional needs, and the need for a durable prosthodontic solution in a medically fragile older adult. The crowns were fixed with a dual‐polymerizing, radiopaque, self‐mixing resin cement, using a self‐etch primer (20‐s application and air‐dry) and a prosthetic primer (Panavia V5 Universal system, Kuraray Noritake, Kuraray Europe GmbH, Germany). After an initial curing time of 5 s per side, the excess cement was removed. The curing process was then completed by applying the curing light for 40 s per surface for each tooth (palatal and buccal, 1000 mW/cm^2^, Valo, Ultradent, Milan, Italy) (Figure 9).

**Figure 8:**
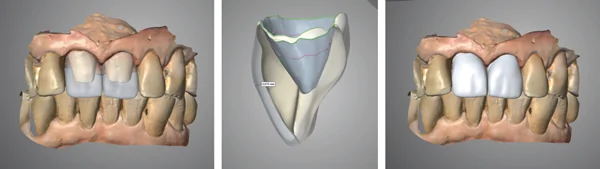
The computer‐aided design (CAD) process for final zirconia crowns.

**Figure 9:**
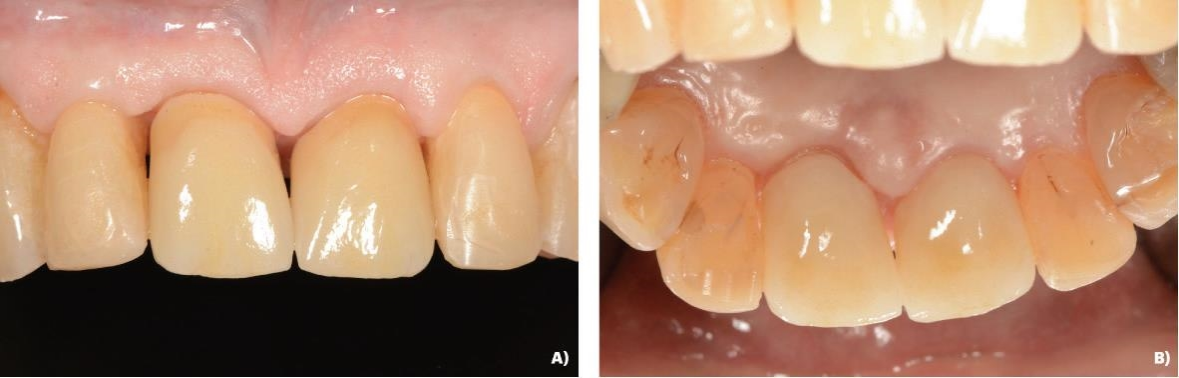
Definitive zirconia crowns on the upper central incisors. (A) Buccal view. (B) Palatal view.

The 2‐year follow‐up shows a stable clinical and radiographic outcome, with excellent maintenance of the gingival profiles (Figure 10). Clinical examination showed stable soft‐tissue contours, absence of symptoms, and satisfactory functional integration, whereas radiographic evaluation revealed stable periapical conditions without evident progression of pathologic changes.

**Figure 10:**
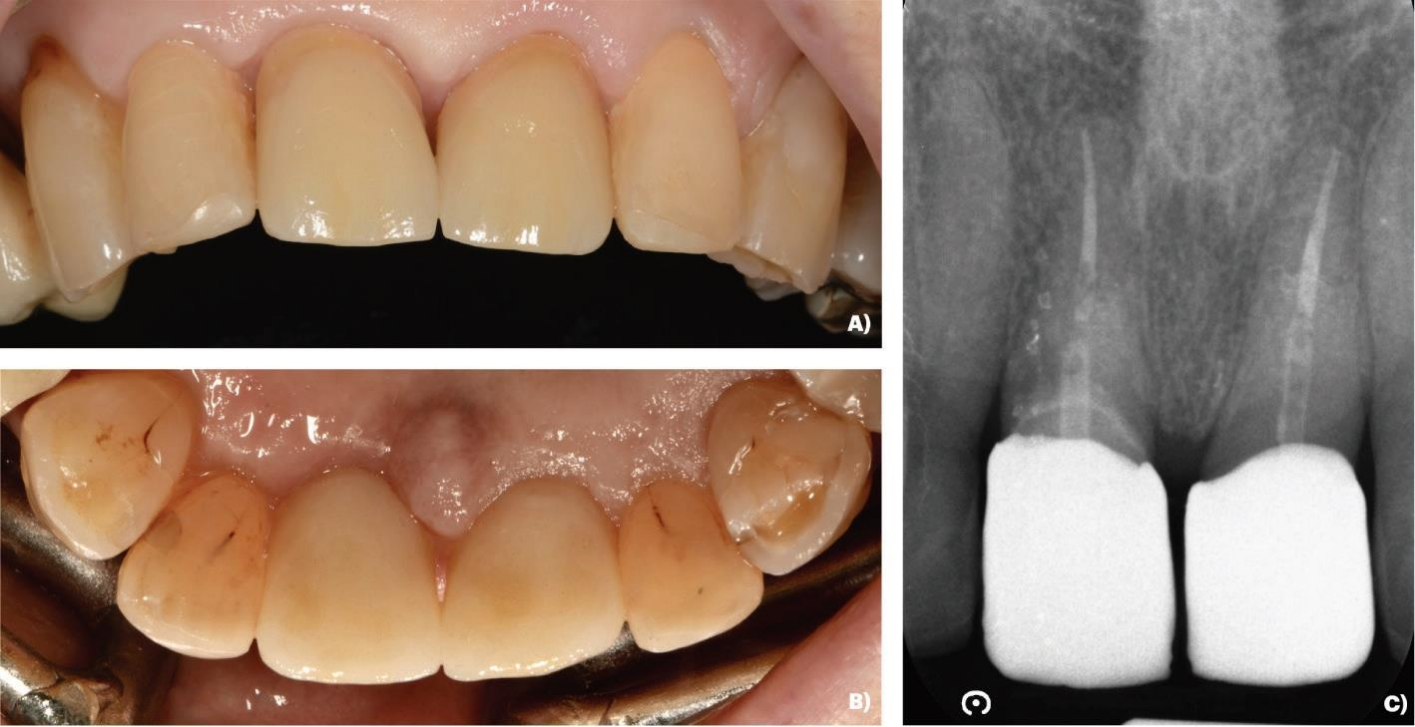
2‐year follow‐up. (A) Buccal view. (B) Palatal view. (C) X‐ray control.

From a patient‐centered perspective, the treatment was well tolerated and allowed recovery of function without reported postoperative complications, an aspect of particular relevance given the patient’s advanced age and overall frailty.

The flowchart summarizing the patient′s treatment and follow‐up is shown in Table 1.

**Table 1:** Flowchart summarizing treatment and follow‐up of the patient.

First access (base line)			**Figure** **1**	Patient presents with maxillary multiple dental trauma. Clinical and radiographic examination. Valuation of dental fractures according to Ellis′s classification.
	Surgery		**Figure** **2**	Tooth 11. Gingivectomy performed using Nd:YAG laser. Bone vaporization obtained with Er:YAG laser. No suture needed.
	Root canal treatment (RCT) and restoration of Tooth 11		**Figure** **3**	Field isolation with rubber dam, RCT with Ni‐Ti rotary instruments. To enhance irrigants′ effect, the SWEEPS technique was applied (Er:YAG laser). Tooth reconstruction with fiber post.

First follow‐up	2 weeks		**Figure** **4**	Complete healing of soft tissue. Late pulpitis of the left central incisor (Tooth 21).

RTC and conservative management of the other teeth impacted		RCT of Tooth 21	**Figure** **5**	RCT with Ni‐Ti rotary instruments. Application of SWEEPS technique to enhance irrigants action.
		Conservative restoration of Teeth 12, 21 and 22	**Figure** **6**	Tooth 21 restoration with fiber post. Teeth 12 and 22 restored with direct composite filling technique.

Prosthetic rehabilitation of Teeth 11 and 21	4 weeks		**Figure** **7**	Vertical finishing preparation was performed, followed by final digital Impression. Fitting of temporary resin crowns.
	5 weeks		**Figure** **8**	Final zirconia crowns designed with CAD (computer‐aided design) process.
			**Figure** **9**	Fitting of definitive zirconia crowns.

Follow‐up	2 years		**Figure** **10**	Stable clinical and radiographic outcome.

## 3. Discussion

Management of TDIs in the elderly poses unique clinical challenges due to frailty, comorbidities, and the frequent use of anticoagulant or antiplatelet therapy. Conventional surgical procedures often carry significant hemorrhagic risk and delay healing. In this context, a minimally invasive, laser‐assisted approach may represent a useful alternative, allowing clinicians to control bleeding, perform selective tissue ablation, and promote wound healing without interrupting systemic medications.

The present case was clinically challenging because treatment planning had to balance trauma severity, advanced age, systemic frailty, and the need to avoid excessive surgical invasiveness under ongoing anticoagulant therapy. In this context, a conservative laser‐assisted approach was selected to support tissue management, bleeding control, and tooth preservation.

In the present case, the different laser wavelengths were not used interchangeably, but for complementary purposes: Nd:YAG for soft‐tissue management and hemostasis, Er:YAG for hard‐tissue ablation and exposure of the fracture margin, and Er:YAG SWEEPS for photoacoustic activation of irrigants during root canal disinfection. This multimodal integration allowed each wavelength to be applied according to its specific tissue interaction profile rather than as a generic adjunct, thereby supporting a minimally invasive and functionally coordinated treatment strategy.

The synergistic use of Nd:YAG, Er:YAG, and Er:YAG SWEEPS lasers provided a coherent and biologically rational workflow. The Nd:YAG laser (1064 nm) ensured precise coagulation and deep decontamination of soft tissues through hemoglobin‐specific absorption, producing a bloodless field even under anticoagulation [17]. The Er:YAG laser (2940 nm), with its high affinity for water and hydroxyapatite, permitted conservative bone contouring and exposure of the fracture margin with minimal thermal impact, preserving surrounding tissues [18, 19]. Finally, the Er:YAG SWEEPS photoacoustic technique enhanced irrigant activation during endodontic disinfection, reinforcing chemomechanical cleaning and biofilm disruption within the root canal system [20, 21]. Together, these wavelengths enabled a coordinated workflow from soft‐tissue control to hard‐tissue preparation and canal decontamination within a minimally invasive treatment strategy.

In comparison with conventional treatment approaches, the multimodal laser‐assisted strategy used in the present case was intended to support local hemostatic control, conservative tissue management, and tooth preservation in a frail anticoagulated patient. Nevertheless, conventional restorative and surgical approaches remain valid, and the present case should be interpreted as an example of a context‐specific minimally invasive option rather than evidence of general superiority.

Beyond the local dimension, facial and dental trauma in frail or medically compromised older adults can trigger significant systemic consequences. Oral pain, infection, and loss of anterior teeth frequently impair feeding ability, speech, and social interaction, leading to reduced nutritional intake, cognitive decline, and psychosocial isolation [22, 23]. Moreover, impaired wound healing and chronic inflammation may serve as portals for bacteraemia, particularly in individuals with cardiac or metabolic vulnerability. Therefore, prompt yet conservative oral rehabilitation is not only a dental need but a key component of multidimensional geriatric care aimed at maintaining systemic stability and quality of life.

In geriatric patients on chronic anticoagulation, the extraction–implant option, although theoretically feasible, is often clinically impractical in the acute setting. Immediate extraction would carry substantial bleeding risk and compromise primary wound closure, whereas early implant placement would be biologically unpredictable due to reduced osteogenic potential, slower vascular response, and impaired osseointegration. Furthermore, suspension of antithrombotic medication—sometimes required for surgical implant placement—could expose fragile geriatric patients to thromboembolic events. In this context, laser‐assisted tooth preservation may represent a conservative, temporizing, and biologically appropriate strategy that maintains alveolar bone and aesthetics while deferring or avoiding further invasive procedures [24, 25].

Favorable soft‐tissue healing was observed within 2 weeks. Although laser‐assisted treatment may have contributed to this outcome through effective hemostatic control, reduced surgical trauma, and improved local decontamination, the specific contribution of photobiomodulatory mechanisms cannot be determined from this single case report. Moreover, the present case primarily involved high‐intensity laser applications for surgical and endodontic purposes rather than a dedicated photobiomodulation protocol; therefore, any interpretation of enhanced healing in photobiomodulatory terms should be made with caution. The ability to achieve effective hemostasis and decontamination without flap elevation may nevertheless be considered consistent with a minimally invasive approach in a patient at high systemic risk. Additionally, the ability to achieve effective hemostasis and disinfection without flap elevation aligns with current recommendations for minimally invasive management in patients at high systemic risk [2].

From a safety perspective, the favorable outcome observed in this case should be interpreted cautiously. In anticoagulated older adults, the potential value of a multimodal laser‐assisted approach lies in its ability to support local hemostatic control and selective tissue management while limiting surgical trauma. However, treatment safety also depends on careful parameter selection, operator experience, and attention to the prevention of thermal injury, postoperative bleeding, and other procedural complications. Therefore, the absence of adverse events in the present case should not be interpreted as evidence that such risks are negligible, but rather as an outcome achieved under controlled clinical conditions.

From a functional standpoint, this integrated laser approach enabled the preservation of the traumatized incisor, avoiding extraction and subsequent prosthetic rehabilitation, which would have been complicated by anticoagulant therapy and bone fragility. The workflow—soft‐tissue conditioning, targeted osteotomy, photoacoustic canal disinfection, adhesive reconstruction, and prosthetic rehabilitation—demonstrates the feasibility of a modern, conservative treatment paradigm consistent with the principles of geriatric and regeneratively oriented dentistry [25, 26].

Despite the favorable 2‐year outcome, the long‐term prognosis of such a severely compromised tooth should remain guarded. Possible complications include loss of periodontal stability, restorative failure, post‐related or root fracture, recurrent endodontic or periapical disease, and the eventual need for extraction if biological or functional conditions deteriorate over time. For this reason, long‐term clinical and radiographic follow‐up remains essential.

Nonetheless, the limitations of this work must be acknowledged. Being a single‐case report, the findings cannot be generalized to all elderly or anticoagulated patients. Moreover, laser‐based protocols require rigorous training, careful parameter selection, and cost considerations. Future multicentre clinical studies and randomized trials should be designed to validate standardized protocols for multilaser applications in complex medically compromised patients.

In conclusion, the present case suggests that multimodal laser therapy (Nd:YAG‐Er:YAG‐SWEEPS) may represent a conservative and biologically respectful treatment option in selected frail anticoagulated patients. Within the limitations of a single case report, this coordinated laser‐assisted workflow supported local control, tooth preservation, and functional rehabilitation over 2 years of follow‐up.

**Nomenclature**
CAD‐CAM: computer‐aided design and computer‐aided manufacturing
EDTA: ethylenediaminetetraacetic acid
Er:YAG: erbium‐doped: yttrium‐aluminum‐garnet
NaOCl: sodium hypochlorite
Nd:YAG: neodymium‐doped: yttrium‐aluminum‐garnet
Ni‐Ti: nickel‐titanium
QSP: quantum square pulse
RCT: root canal treatment
SWEEPS: shock wave‐enhanced emission photoacoustic streaming
TDI/TDIs: traumatic dental injury/traumatic dental injuries
WL: working length
WSES: World Society of Emergency Surgery

## Author Contributions

**Ilaria Giovannacci:** conceptualization, investigation, data curation, writing – original draft, writing – review & editing. **Beatrice Spaggiari:** investigation, data curation, writing – review & editing. **Rita Antonelli:** investigation, writing – review & editing. **Marco Meleti:** supervision, writing – review & editing. **Paolo Vescovi:** conceptualization, supervision, writing – review & editing.

## Funding

Open access publishing was facilitated by the Universita degli Studi di Parma, as part of the Wiley‐ CRUI‐CARE agreement.

## Disclosure

All authors have read and approved the final version of the manuscript. Corresponding Author (Ilaria Giovannacci) had full access to all of the data in this study and takes complete responsibility for the integrity of the data and the accuracy of the data analysis.

## Ethics Statement

This is a single anonymized case report with informed consent. The treatment was carried out in accordance with the principles of the Declaration of Helsinki.

## Consent

Written informed consent for publication of their clinical details and/or clinical images was obtained from the patient.

## Conflicts of Interest

The authors declare no conflicts of interest.

## Data Availability

Data sharing is not applicable to this article as no datasets were generated or analysed during the current study.

## References

[1] Brucoli M. , Boffano P. , Romeo I. , Corio C. , Benech A. , Ruslin M. , Forouzanfar T. , Starch-Jensen T. , Rodríguez-Santamarta T. , De Vicente J. C. , Snäll J. , Thorén H. , Aničić B. , Konstantinovic V. S. , Pechalova P. , Pavlov N. , Daskalov H. , Doykova I. , Kelemith K. , Tamme T. , and Dediol E. , Epidemiology of Maxillofacial Trauma in the Elderly: A European Multicenter Study, Journal of Stomatology Oral and Maxillofacial Surgery. (2020) 121, no. 4, 330–338, 10.1016/j.jormas.2019.09.002, 31533064. 31533064

[2] de Simone B. , Chouillard E. , Podda M. , Pararas N. , de Carvalho Duarte G. , Fugazzola P. , Birindelli A. , Coccolini F. , Polistena A. , Sibilla M. G. , Kruger V. , Fraga G. P. , Montori G. , Russo E. , Pintar T. , Ansaloni L. , Avenia N. , Di Saverio S. , Leppäniemi A. , Lauretta A. , and Catena F. , The 2023 WSES Guidelines on the Management of Trauma in Elderly and Frail Patients, World Journal of Emergency Surgery. (2024) 19, no. 1, 10.1186/s13017-024-00537-8, 38816766. PMC11140935 38816766

[3] de Andrade F. B. , Lebrão M. L. , Santos J. L. , and Duarte Y. A. , Relationship Between Oral Health and Frailty in Community-Dwelling Elderly Individuals in Brazil, Journal of the American Geriatrics Society. (2013) 61, no. 5, 809–814, 10.1111/jgs.12221, 23647172. 23647172

[4] Dros C. , Sealy M. J. , Krijnen W. P. , Weening-Verbree L. F. , Hobbelen H. , and Jager-Wittenaar H. , Oral Health and Frailty in Community-Dwelling Older Adults in the Northern Netherlands: A Cross-Sectional Study, International Journal of Environmental Research and Public Health. (2022) 19, no. 13, 10.3390/ijerph19137654, 35805314. PMC9265776 35805314

[5] Slashcheva L. D. , Karjalahti E. , Hassett L. C. , Smith B. , and Chamberlain A. M. , A Systematic Review and Gap Analysis of Frailty and Oral Health Characteristics in Older Adults: A Call for Clinical Translation, Gerodontology. (2021) 38, no. 4, 338–350, 10.1111/ger.12577, 34331353. 34331353

[6] Uskur T. and Kurt B. F. , Polypharmacy and Drug-Drug Interactions in Elderly Patients With Gastrointestinal Bleeding: A Single-Center Retrospective Study, Cureus. (2025) 17, no. 1, e77866, 10.7759/cureus.77866, 39991352. PMC11847164 39991352

[7] Tanya S. , Prajaneh S. , Patcharanuchat P. , and Sattayut S. , Laser-Mediated Hemostasis for Older Patients Receiving Routine Dental Treatment, Dentistry Journal. (2025) 13, no. 7, 10.3390/dj13070315, 40710160. PMC12293330 40710160

[8] DiVito E. , Peters O. A. , and Olivi G. , Effectiveness of the Erbium:YAG Laser and New Design Radial and Stripped Tips in Removing the Smear Layer After Root Canal Instrumentation, Lasers in Medical Science. (2012) 27, no. 2, 273–280, 10.1007/s10103-010-0858-x, 21120568. 21120568

[9] Imtiaz T. , Adimulapu H. S. , Pandiyan I. , and Bhandari S. , Dentist Perspective on Knowledge and Practice Toward Ellis Class 3 Tooth Fracture: A Survey, Journal of Advanced Pharmaceutical Technology & Research. (2022) 13, no. Supplement 2, S387–S391, 10.4103/japtr.japtr_393_22, 36798551. PMC9926606 36798551

[10] Lukac M. , Primc N. , and Pirnat S. , Quantum Square Pulse Er:YAG Lasers for Fast and Precise Hard Dental Tissue Preparation, Laser and Health Academy. (2012) 1, no. 2, 14–21.

[11] Gomes B. P. F. A. , Aviero E. , and Kishen A. , Irrigants and Irrigation Activation Systems in Endodontics, Brazilian Dental Journal. (2023) 34, no. 4, 1–33, 10.1590/0103-6440202305577, 37909632. PMC10642269 37909632

[12] Fiegler-Rudol J. , Grzech-Leśniak Z. , Tkaczyk M. , Grzech-Leśniak K. , Zawilska A. , and Wiench R. , Enhancing Root Canal Disinfection With Er:YAG Laser: A Systematic Review, Dentistry Journal. (2025) 13, no. 3, 10.3390/dj13030101, 40136729. PMC11941447 40136729

[13] Nugala B. , Santosh Kumar B. B. , Sahitya S. , and Mohana Krishna P. , Biologic Width and Its Importance in Periodontal and Restorative Dentistry, Journal of Conservative Dentistry. (2012) 15, no. 1, 12–17, 10.4103/0972-0707.92599, 22368328. PMC3284004 22368328

[14] Kois J. C. , Altering Gingival Levels: The Restorative Connection Part I: Biologic Variables, Journal of Esthetic and Restorative Dentistry. (1994) 6, no. 1, 3–7, 10.1111/j.1708-8240.1994.tb00825.x.

[15] Mulla S. A. , Patil A. , Mali S. , Jain A. , Sharma D. , Jaiswal H. C. , Saoji H. A. , Jakhar A. , Talekar S. , and Singh S. , Exploring the Biological Width in Dentistry: A Comprehensive Narrative Review, Cureus. (2023) 15, no. 7, e42080, 10.7759/cureus.42080, 37602053. PMC10434820 37602053

[16] Rathaur S. , Gupta P. K. , Dhote S. , Pravin K. S. , Kishlay K. , and Gupta S. , Effect of Ferrule Height on the Fracture Resistance of Endodontically Treated Teeth Restored With Glass Fiber Posts: An In Vitro Study, Cureus. (2025) 17, no. 2, e79583, 10.7759/cureus.79583, 40151713. PMC11946697 40151713

[17] Costa-Tort J. , Schiavo-Di Flaviano V. , González-Navarro B. , Jané-Salas E. , Estrugo-Devesa A. , and López-López J. , Update on the Management of Anticoagulated and Antiaggregated Patients in Dental Practice: Literature Review, Journal of Clinical and Experimental Dentistry. (2021) 13, no. 9, e948–e956, 10.4317/jced.58586, 34603625. PMC8464381 34603625

[18] Vescovi P. , De Francesco P. , Giovannacci I. , Leão J. C. , and Barone A. , Piezoelectric Surgery, Er:YAG Laser Surgery and Nd:YAG Laser Photobiomodulation: A Combined Approach to Treat Medication-Related Osteonecrosis of the Jaws (MRONJ), Dentistry Journal. (2024) 12, no. 8, 10.3390/dj12080261, 39195105. PMC11353968 39195105

[19] Giovannacci I. , Giunta G. , Pedrazzi G. , Meleti M. , Manfredi M. , Migliario M. , Brucoli M. , Greco Lucchina A. , Mortellaro C. , and Vescovi P. , Erbium Yttrium-Aluminum-Garnet Laser Versus Traditional Bur in the Extraction of Impacted Mandibular Third Molars: Analysis of Intra- and Postoperative Differences, Journal of Craniofacial Surgery. (2018) 29, no. 8, 2282–2286, 10.1097/SCS.0000000000004574, 29742567. 29742567

[20] Bago I. , Durin A. , Kanizaj D. , Vuletić L. B. , Vidović Zdrilić I. , and Anić I. , The Efficacy of a Novel SWEEPS Laser-Activated Irrigation Compared to Ultrasonic Activation in the Removal of Pulp Tissue From an Isthmus Area in the Apical Third of the Root Canal, Lasers in Medical Science. (2023) 38, no. 1, 10.1007/s10103-023-03857-4, 37599293. 37599293

[21] Bao P. , Liu H. , Yang L. , Zhang L. , Yang L. , Xiao N. , Shen J. , Deng J. , and Shen Y. , In Vitro Efficacy of Er:YAG Laser-Activated Irrigation Versus Passive Ultrasonic Irrigation and Sonic-Powered Irrigation for Treating Multispecies Biofilms in Artificial Grooves and Dentinal Tubules: An SEM and CLSM Study, BMC Oral Health. (2024) 24, no. 1, 10.1186/s12903-024-04042-x, 38389109. PMC10882935 38389109

[22] Rapp L. , Sourdet S. , and Lacoste-Ferré M. H. , Oral Health and Undernutrition in the Frail Elderly Persons, Journal of Nutrition, Health and Aging. (2021) 25, no. 4, 484–491, 10.1007/s12603-020-1546-6, 33786566. PMC12876686 33786566

[23] Rai S. , Misra D. , Misra A. , Jain A. , and Bisla S. , Impact of Oral Health Factors on Quality of Life of Geriatric Population - A Systematic Review, Journal of Indian Academy of Oral Medicine and Radiology. (2021) 33, no. 4, 10.4103/jiaomr.jiaomr_98_20.

[24] van Ginkel D. J. , Bor W. L. , Aarts H. M. , Dubois C. , De Backer O. , Rooijakkers M. J. P. , Rosseel L. , Veenstra L. , van der Kley F. , van Bergeijk K. H. , Van Mieghem N. M. , Agostoni P. , Voskuil M. , Schotborgh C. E. , IJsselmuiden A. J. J. , Van Der Heyden J. A. S. , Hermanides R. S. , Barbato E. , Mylotte D. , Fabris E. , Frambach E. , Dujardin P. , Ferdinande K. , Peper B. , Rensing J. , Timmers B. J. W. M. , Swaans L. , Brouwer M. J. , Nijenhuis J. , Overduin V. J. , Adriaenssens D. C. , Kobari T. , Vriesendorp Y. , Montero-Cabezas P. A. , El Jattari J. M. , Halim H. , Van den Branden J. , Leonora B. J. L. , Vanderheyden R. , Lauterbach M. , Wykrzykowska M. , Hof J. J. v. ′t. , van Royen A. W. J. , Tijssen N. , Delewi J. G. P. , Ten Berg R. , and J M & POPular PAUSE TAVI Investigator , Continuation Versus Interruption of Oral Anticoagulation During TAVI, New England Journal of Medicine. (2025) 392, no. 5, 438–449, 10.1056/NEJMoa2407794, 39216096. 39216096

[25] Tabowei G. , Dadzie S. K. , Ahzam R. M. , Rehman M. M. , Blair K. , Habib I. , Wei C. R. , and Amin A. , Continuation Versus Interruption of Anticoagulation During Transcatheter Aortic Valve Implantation: A Systematic Review and Meta-Analysis, Cureus. (2024) 16, no. 12, e76434, 10.7759/cureus.76434, 39867032. PMC11763579 39867032

[26] Diz P. , Scully C. , and Sanz M. , Dental Implants in the Medically Compromised Patient, Journal of Dentistry. (2013) 41, no. 3, 195–206, 10.1016/j.jdent.2012.12.008. 23313715

